# Evaluation of variant calling tools for large plant genome re-sequencing

**DOI:** 10.1186/s12859-020-03704-1

**Published:** 2020-08-17

**Authors:** Zhen Yao, Frank M. You, Amidou N’Diaye, Ron E. Knox, Curt McCartney, Colin W. Hiebert, Curtis Pozniak, Wayne Xu

**Affiliations:** 1 Morden Research and Development Centre, Agriculture and Agri-Food Canada, 101 Route 100, Morden, Manitoba R6M 1Y5 Canada; 2 Ottawa Research and Development Centre, Agriculture and Agri-Food Canada, 960 Carling Avenue, Ottawa, Ontario K1A 0C6 Canada; 3 Department of Plant Sciences, University of Saskatchewan, Saskatoon, Saskatchewan S7N 5A8 Canada; 4 Swift Current Research and Development Centre, Agriculture and Agri-Food Canada, Box 1030, Swift Current, Saskatchewan S9H 3X2 Canada

**Keywords:** Variant calling, Sequence mapping tools, Variant calling tools, Tool evaluation, Wheat exome capture sequences

## Abstract

**Background:**

Discovering single nucleotide polymorphisms (SNPs) from agriculture crop genome sequences has been a widely used strategy for developing genetic markers for several applications including marker-assisted breeding, population diversity studies for eco-geographical adaption, genotyping crop germplasm collections, and others. Accurately detecting SNPs from large polyploid crop genomes such as wheat is crucial and challenging. A few variant calling methods have been previously developed but they show a low concordance between their variant calls. A gold standard of variant sets generated from one human individual sample was established for variant calling tool evaluations, however hitherto no gold standard of crop variant set is available for wheat use. The intent of this study was to evaluate seven SNP variant calling tools (FreeBayes, GATK, Platypus, Samtools/mpileup, SNVer, VarScan, VarDict) with the two most popular mapping tools (BWA-mem and Bowtie2) on wheat whole exome capture (WEC) re-sequencing data from allohexaploid wheat.

**Results:**

We found the BWA-mem mapping tool had both a higher mapping rate and a higher accuracy rate than Bowtie2. With the same mapping quality (MQ) cutoff, BWA-mem detected more variant bases in mapping reads than Bowtie2. The reads preprocessed with quality trimming or duplicate removal did not significantly affect the final mapping performance in terms of mapped reads. Based on the concordance and receiver operating characteristic (ROC), the Samtools/mpileup variant calling tool with BWA-mem mapping of raw sequence reads outperformed other tests followed by FreeBayes and GATK in terms of specificity and sensitivity. VarDict and VarScan were the poorest performing variant calling tools with the wheat WEC sequence data.

**Conclusion:**

The BWA-mem and Samtools/mpileup pipeline, with no need to preprocess the raw read data before mapping onto the reference genome, was ascertained the optimum for SNP calling for the complex wheat genome re-sequencing. These results also provide useful guidelines for reliable variant identification from deep sequencing of other large polyploid crop genomes.

## Background

Recent advances in next generation sequencing (NGS) technology enables us to detect genome wide genetic variants, such as single nucleotide polymorphisms (SNPs) and insertion/deletions (INDELs) at a low cost. There are three basic approaches for generating sequence data for genome wide variant detection against a genome reference including whole genome sequencing (WGS), genotype-by-sequencing (GBS), and whole exome capture (WEC) sequencing, each with different strengths and applications. WGS covers the whole genome including the large non-coding genomic sequence regions [1, 2] while WEC focuses on the coding exome [3, 4]. GBS applies specific restriction enzymes for genome reduction followed by barcoding samples, and works for both large and small genomes [5–10]. Because some genetic variants are associated with gene function, the WEC analysis would be more immediately relevant to the interpretation of variants underlying trait variation [11].

With high coverage of short sequence reads generated from the same regions of a crop genome, the first task is to align these sequence reads to corresponding regions of a reference genome. Currently, more than 60 different algorithms exist for mapping sequence reads to a reference genome [12, 13]. These mapping tools use different algorithms to ensure that the short sequence reads are aligned accurately and quickly to the correct locations of the reference genome. The mapped read number with metrics of specificity and sensitivity as well as the mapping time can be used to evaluate the mapping tools appropriate for a specific reference genome [13]. Several studies have compared different mapping tools for either genomic sequence or RNA-seq data [13–19]. Their conclusions are similar in that all different mapping tools have a big overlap with the same reads mapped to the same loci [19]. The remaining differently mapped or unmapped reads among the variety of tools were ascribed to the various parameters of experiments such as sequencing platforms, genome complexity, and sequence quality [13]. Once short read sequences are aligned to the reference genome, variant calling tools determine if a SNP or an INDEL exists in the alignment. Many variant calling software tools have been developed in recent years. Performance comparison of different variant calling tools has been conducted in the diploid human genome and polyploid crop genomes [20–22]. Surprisingly there was substantial disagreement among variant calls made by different variant calling tools/pipelines in several studies [23, 24]. Polyploid crop genomes, for example wheat (*Triticum aestivum* L., 2n = 6x = 42, allohexaploid), magnify the complexity and challenges in both sequence mapping and variant detection.

The intent of this study was to find the variant calling tools that are most suitable for wheat by evaluating different mapping tools and variant calling tools. As previous studies pointed out that sequence quality may impact the sequence mappers’ performances, here we focused on the two popular mapping tools, BWA-mem [25] and Bowtie2 [26] considering differently preprocessed sequence data from the same sequence platform and the same genome, instead of repeating the similar comparison approaches as in previous reports. Then we further evaluated seven variant calling tools on the mapped data including the Genome Analysis Tool Kit (GATK) [27], Samtools/mpileup [28], FreeBayes [29], Platypus [30], SNVer [31], VarDict [32], and VarScan [33]. These tools are widely employed in genomic variant analyses and many of the algorithms used in these variant calling tools were originally developed and evaluated in human genome sequence studies, yet are frequently used in plant genomic research [22].

In order to compare the performance of different variant calling tools, the Genome in a Bottle (GIAB) consortium [34] has developed high confidence variant sets (true positives) from one human individual and then generated several sequence data sets from the same individual by different next generation sequencing (NGS) technologies. These data sets serve as a gold standard for variant calling tool comparison for human genetic variant discovery [21, 35, 36]. The high confidence variant sets allow the estimation of true positive rate (TPR) and false positive rate (FPR) of different calling tools. Since there is no gold standard of crop variant sets available, we defined a “true” positive SNP list as those calls that were supported by multiple variant calling tools. We compared these seven variant calling tools based on concordance and area under the curve of receiver operating characteristic (ROC) [37].

## Results

### Mapping tool comparisons using differently pre-processed sequence data

**Fig. 1:**
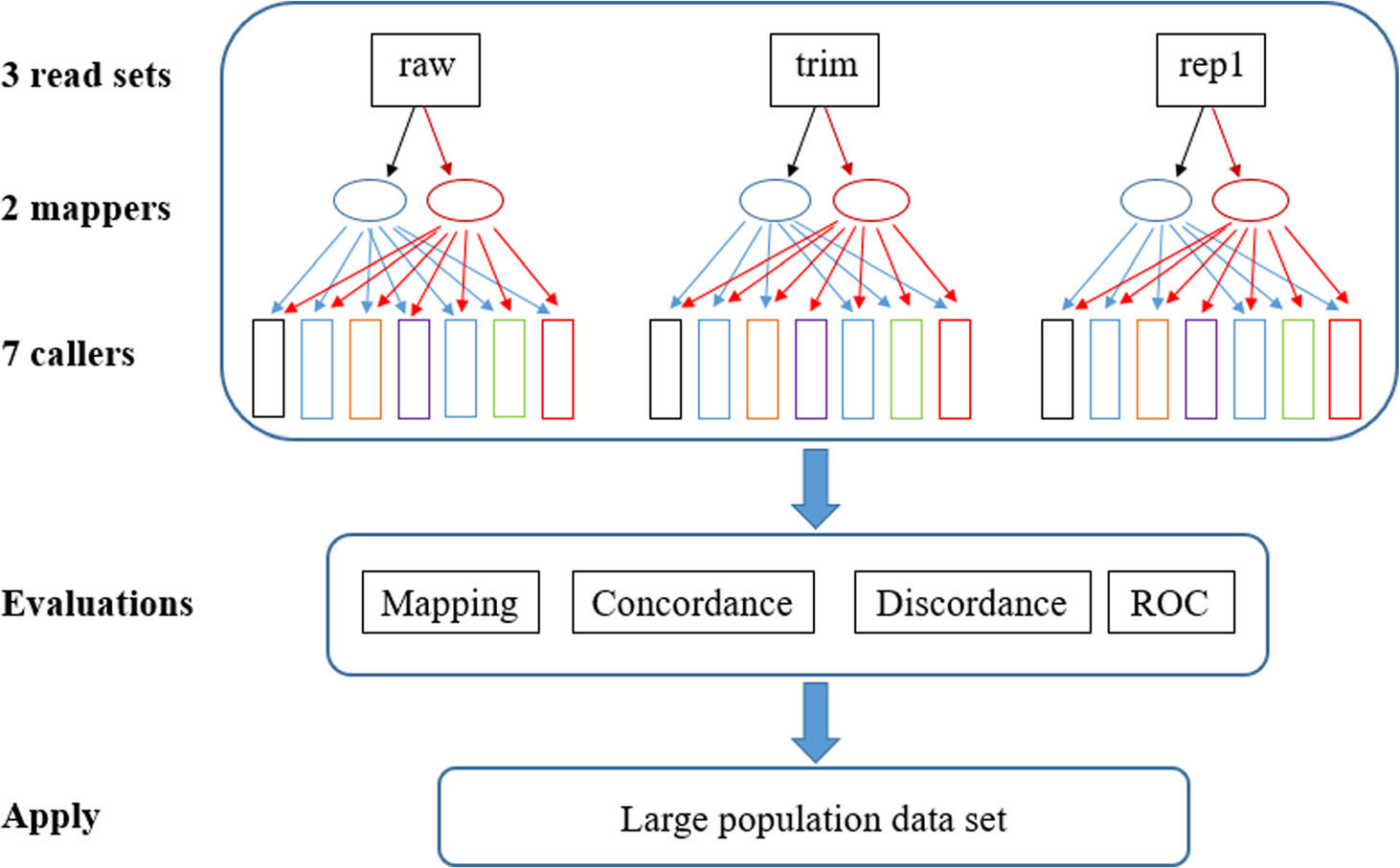
Experimental design. A raw read set in fastq (raw) and two preprocessed sets by quality trimming (trim) and duplicate removal (rep1) are shown in the first row of rectangles. Each set was aligned to the wheat reference genome v1.0 by one of two mapping tools (mappers): BWA-mem or Bowtie2. The mapped bam files were used for variant calling using seven variant tools (callers). A total of 42 combinations/experiments were evaluated for two mapping tools and seven variant calling tools (callers) using three data preprocess types by criteria: Mapping, Concordance, Discordance and Receiver Operating Characteristic (ROC). The final settings were applied to a large population data set

**Fig. 2:**
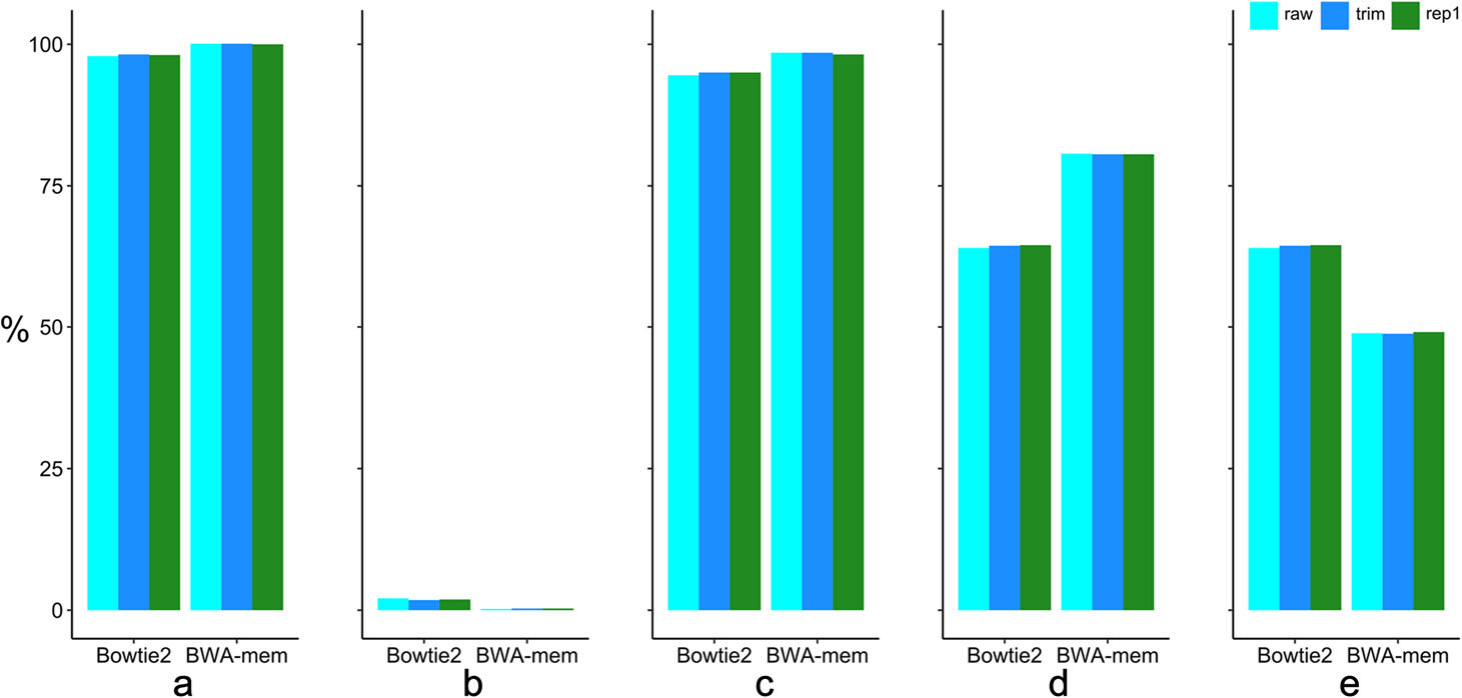
Comparison of mapping tools. The raw sequence read fastq file (raw) and the preprocessed trimmed (trim), or duplicate removal (rep1) fastq files were mapped onto wheat reference genome v1.0 by Bowtie2 or BWA-mem. The mapping statistics were calculated by Samtools using different flags. The Y-axis represents the percentage of read counts among total raw reads. **a**. The percentage of total mapped reads in six different experiments (two **mapping tool**s and three data preprocesses) among total raw read number. **b**. The percentage of unmapped read number. **c**. The percentage of properly paired mapped reads whose reads R1 and R2 from the same segment are mapped on the same chromosome at a good expected distance with the correct directions. **d**. The percentage of read number greater than mapping quality (MQ) of 10. **e**. The percentage of mapped read numbers that had MQ greater than 10 and mismatched bases less than 10 per read

A WEC sequence data set was used from allohexaploid wheat. A total of 97,280,936 WEC 100 bp paired-end reads (**Supplementary Table** **S1**) were generated from one wheat line. 96,718,760 reads were retained after quality trimming and 70,099,964 after duplicate removal. Given that the WEC data set includes a total of 321 Mb sequences [3], the three read sets had a coverage depth of ~30x, ~30x, or ~ 23x. Following the experimental design in Fig. 1, we obtained the total mapped reads, the rate of the properly mapped paired-end reads, map quality (MQ) greater or equal to 10, and mismatched bases per read less than 10 (Fig. 2a-e). In the raw and quality trimmed read data, BWA-mem mapped more reads (100.1%) than the total number of reads (**Supplementary Table** **S1****)**, with a small number of unmapped reads. Bowtie2 obtained 98% mapping rate with more unmapped reads than BWA-mem. BWA-mem had a higher mapping rate (98.5%) than Bowtie2 (94.5%) for properly mapped paired-end reads whose reads R1 and R2 generated from the same segment were mapped on the same chromosome at a good expected distance with the correct directions.

Furthermore, more mapped reads passed the quality filtering in BWA-mem than Bowtie2 (Fig. 2d). Bowtie2 had more reads than BWA-mem in less than 10 mismatched bases per read (Fig. 2e); accordingly, BWA-mem had more reads that contained more than 10 mismatched bases per read, which suggests BWA-mem may catch more variants than Bowtie2.

After the sequence read quality was improved by removing low base quality (trim) and duplicate (rep1) reads (Fig. 2**& Supplementary Table** **S1****)**, total mapped reads dropped accordingly and the properly paired mapped reads number were still lower than raw reads mapping. These results suggest that the quality filtering by both base quality and duplicates lowered the mapping performance in both BWA-mem and Bowtie2 in terms of mapped reads. After bad quality bases were trimmed off from both ends of sequence reads, the mapping performances were very close to the raw sequence reads mapping of BWA-mem and Bowtie2.

### Concordance of the seven variant calling tools

The concordances of the seven variant calling tools (Table 1**)** were assessed in a total of 42 experiments. To make fair comparisons between different variant calling tools, we applied the same filtering to all VCF files of average variant quality score (QUAL) greater than 5 and minimum total number of reads containing this variant (TR) of 3 (QUAL / TR > 5 & TR > 2) (**Supplementary Data** **1****,** **2****,** **3****,** **4****,** **5****,** **6****,** **7****)**. This filter setting was focused on the actual TRs and their average call quality. After this filtering, the numbers of variants identified from the seven tools on each of the two mapping tools and different preprocessed read data sets ranged from 160,000 to 1.8 million (**Supplementary Table** **S2****)**. With the raw sequence reads, FreeBayes from BWA-mem mapping called the highest number (1,792,861) of SNPs while Platypus with Bowtie2 called the lowest number (166,063). For the 321 Mb target sequences determined by the WEC protocol [3], approximately 1 million SNPs were estimated, given that ~ 1 SNP exists per 300 bases. Forty-two variant call format (vcf) files were obtained using seven variant calling tools and two read mapping tools on three types of preprocessed data sets from the same sequencing sample and 2,397,343 unique SNPs were observed.

**Table 1:** Algorithms and short descriptions of the seven variant calling tools

Variant tool	Version	Algorithm	Pipelines	Default filter	Reference
FreeBayes	v1.2.0–2	Haplotype-based	FreeBayes	^b^10,^m^1	Garrison E, et al, 2012 [29]
		Bayesian			
GATK	4.0.11.0	Haplotype-based	MarkDuplicates	^b^10,^m^20	DePristo M, et al, 2011 [27]
		significant test	BaseRecalibrator		
			HaplotypeCaller		
Platypus	0.8.1	Haplotype-based	Platypus callVariants	^b^20,^m^20	Rimmer A, et al, 2014 [30]
		significant test			
Samtools /mpileup	1.9	Site align-based	Samtools/mpileup	^b^13,^m^0	Li H, 2011 [28]
		gt likelihoods	bcftools call		
SNVer	0.5.3	Site align-based	SNVerIndividual	^b^17,^m^20	Wei Z, et al, 2011 [31]
		MAF *p*-value		^f^0.25,^r^1,^*p*^0.05	
VarScan	v2.3.9	Site-based	Samtools/mpileup	^b^15,^m^0	Koboldt D, et al, 2012 [33]
		allele frequency	mpileup2snp	^f^0.2,^r^2,^*p*^0.01	
VarDict	2018	Site-based	VarDict	^b^22.5,^m^0	Lai Z, et al, 2016 [32]
		alleles Fisher’s	var2vcf_valid	^f^0.01,^r^2	

^a^Only default settings were listed. ^b^BQ Base quality; ^m^MQ Mapping quality; ^r^VR Variant containing reads or total reads containing variants (TR); ^f^VF Variant frequency; ^*p*^ P-value; ^d^DP Depth coverage

**Fig. 3:**
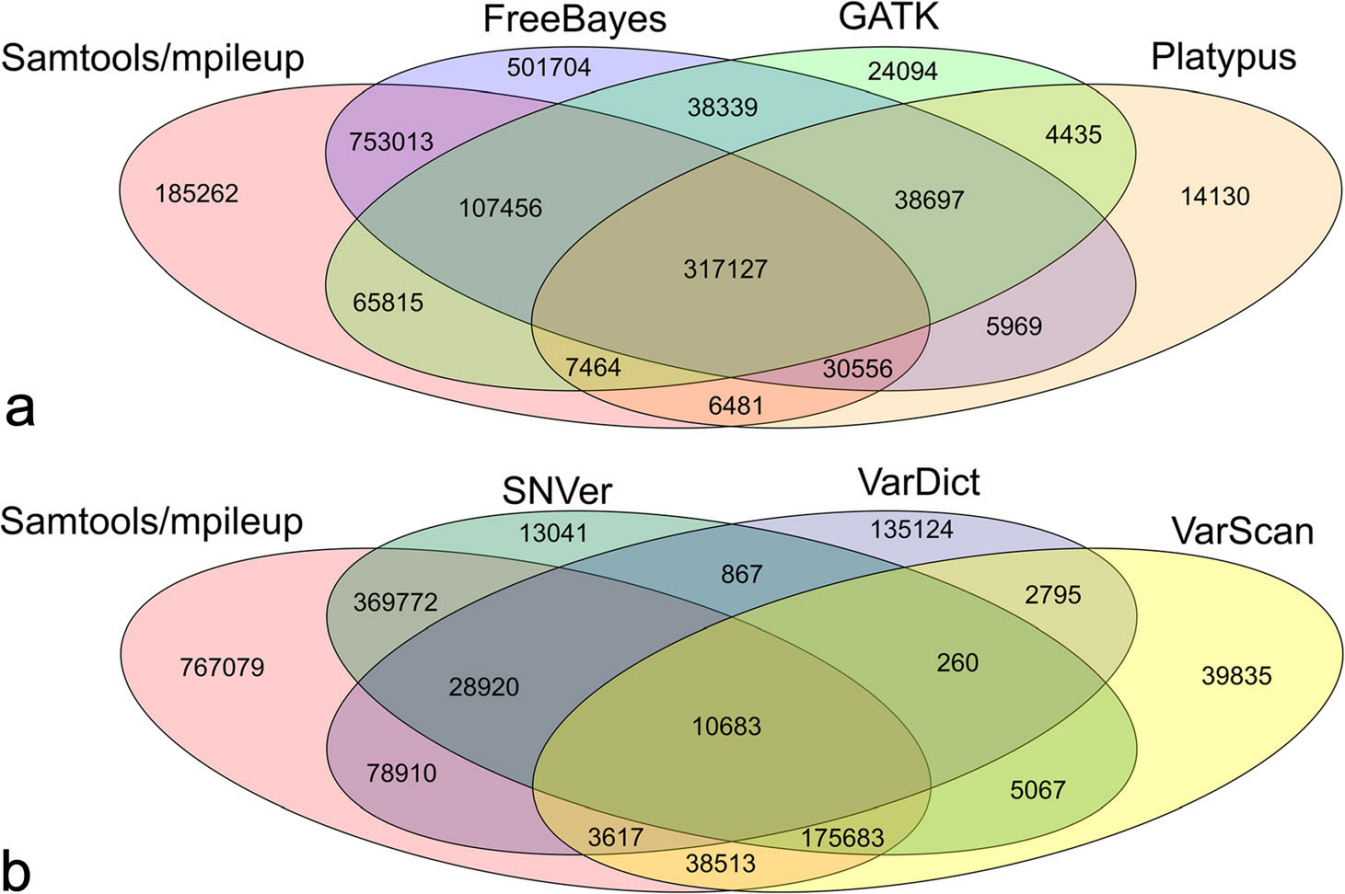
Venn diagrams for variant calling tool comparison. SNP variants were called using different variant calling tools and filtered through the same stringent filtering criteria. The numbers of overlap and unique SNP loci were displayed. **a**. Samtools/mpileup compared with FreeBayes, GATK, and Platypus. **b** Samtools/mpileup compared with SNVer, VarDict, and VarScan

**Fig. 4:**
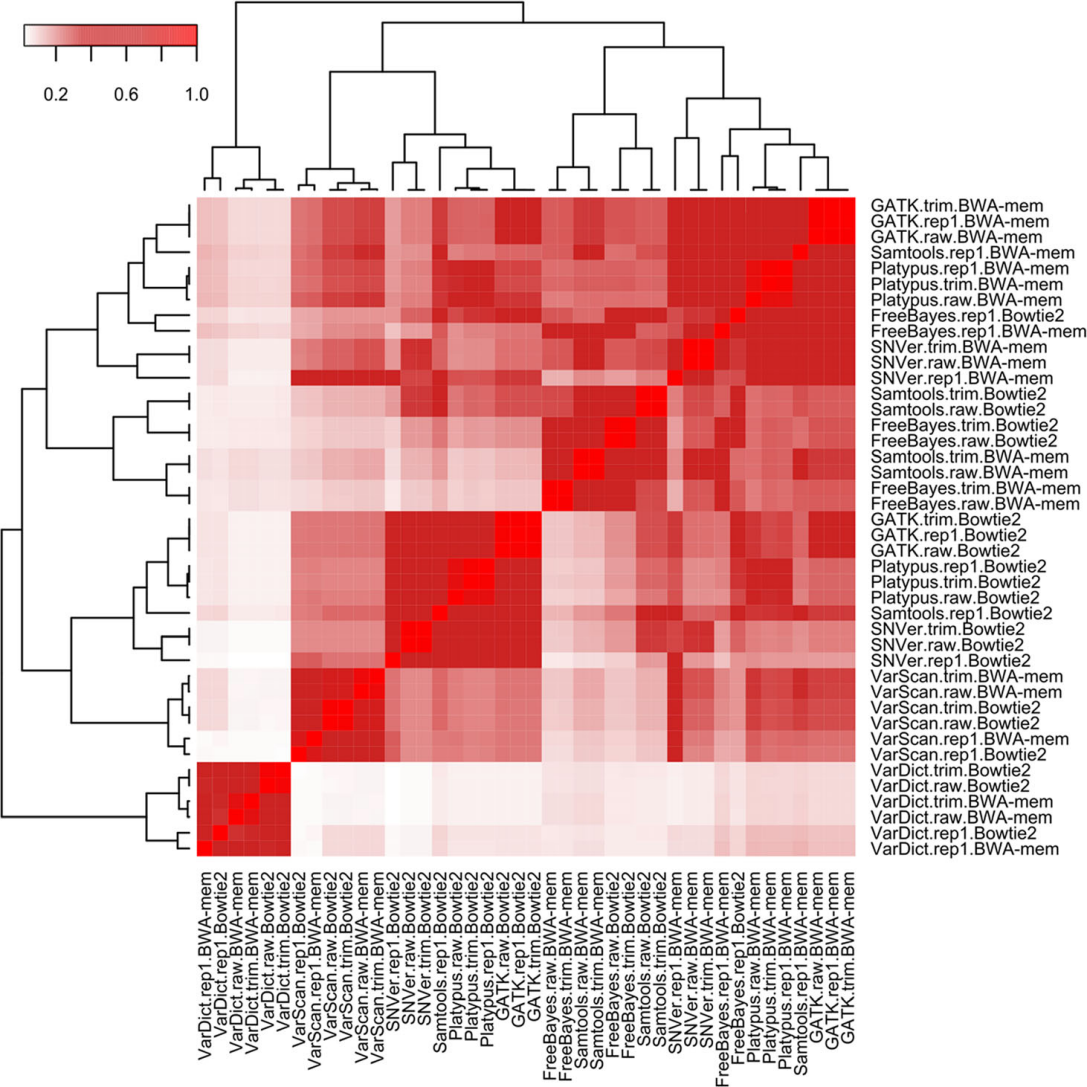
Similarity heatmap of variant calling tools. The SNP call similarity between two variant calling tools was calculated by a percentage of SNP calls shared by two tools. The darker color represents a higher similarity between the paired variant calling tools or their neighbors

When we first examined the SNP concordance of seven variant calling tools on the same preprocessed read sets and the same mapping tool there were only a few common SNPs (8432) called by all seven tools from the same bam file (raw reads mapped by BWA-mem). However, the results of some tools were closer than others. Samtools/mpileup had more overlapping SNP calls (317,127) (Fig. 3a**)** with GATK, FreeBayes, and Platypus than with SNVer, VarScan, and VarDict (10,683) (Fig. 3b**)**. When the pair-wise concordance similarity was calculated by a percentage of the common variant calls over all SNPs, five clusters were identified (Fig. 4), corresponding to VarDict (Cluster 1), VarScan (Cluster 2), SNVer + Samtools/mpileup + Platypus + GATK (Cluster 3), FreeBayes + Samtools/mpileup (Cluster 4), and GATK + Platypus + FreeBayes + SNVer (Cluster 5). The unique VarDict cluster was distinct from other variant calling tools.

The impacts of trimming and duplicate removal on the number of variants called were evaluated. We did not pool all 42 datasets into three categories (raw, trim, rep1) because the impacts could also partly come from the different mapping and calling methods in each category. We investigated the SNP concordance of the three different preprocessed read sets with the same variant calling tool and the same mapping tool (Table **2**, **Supplementary Table** **S2**). For example, the data preprocessing methods affected the outcomes generated by the Samtools/mpileup tool (**Supplementary Fig.** **S1****)**. Three data sets of raw sequence reads, duplicate removal, and quality trimming, showed high concordance in GATK and Platypus calling. Other variant calling tools only had ~ 50% concordance on the same preprocessed read sets (Fig. 4).

**Table 2:** Missed and unique calls for seven variant calling tools to show discordance

		FreeBayes	GATK	Samtools	Platypus	SNVer	VarScan	VarDict
**Missed calls**								
Bowtie2	raw	105	73	397	295	521	4000	52,215
	trim	98	79	382	281	526	4146	52,885
	rep1	43	54	204	148	316	5179	45,663
BWA-	raw	254	354	100	1051	1035	14,773	120,053
mem	trim	249	370	84	1044	1073	15,334	120,792
	rep1	159	214	47	677	860	20,054	97,722
BWA_raw loci	chr2B93040165	missed	868, 32	225, 47	1019, 37	67	47	230, 66
	chr7D397560208	67, 7	29,4 (f)	94, 7	71,4	7	7	112, 7
	chr2A36897168	220,27	323, 18	44, 36 (f)	457, 21	26	36	200, 45
	chr1A14084557	260, 46	903, 25	134, 45	missed	51	45	207, 42
	chr3B133439650	122, 8	71, 3	114, 8	147, 3	missed	8	120, 8
	chr1A11663841	150,5	79,4	110,4	117,5	5	missed	79,4
	chr1A42695010	336,16	71, 4	152,9	54,9	16	9	missed
**Unique calls**								
Bowtie2	raw	398,709	3568	33,865	1516	1738	124,696	170,880
	trim	389,184	3601	33,522	1790	1688	124,154	170,837
	rep1	185,984	5580	9162	1477	608	78,446	109,310
BWA-mem	raw	472,999	20,057	134,717	6614	4429	30,120	102,023
	trim	447,527	20,397	132,482	7411	4283	29,518	100,868
	rep1	167,601	32,299	36,730	6532	1569	15,526	55,835
BWA_raw loci	chr1A23072417	111,4	21,2	59,2	none	none	none	none
	chr2A178387501	none	101,3	63,2	none	none	none	none
	chr1A286123173	none	none	95,4	none	none	none	none
	chr1A357077575	none	none	none	35,3	none	none	none
	chr7D310148693	6,23	none	10,11	none	19	none	none
	chr1A232066481	none	none	none	none	none	6	none
	chr1B23898050	none	none	none	none	none	none	73, 4

The missed calls were only called by one of the seven variant calling tools. The total missed call numbers of seven variant calling tools using either Bowtie2 or BWA-mem mapping and seven locus examples (five “missed” SNP calls and two falsely missed calls due to filtering) were presented. The falsely missed loci were indicated by call QUAL, total read depth, filtered (score, depth, and f). Other calls were presented by call QUAL score and total read depth (score, and depth). SNVer and vascan did not have QUAL score. The unique calls appeared in only one out of the seven variant calling tools. Total unique calls and seven locus examples were presented. The loci were not called with “none”, or called with QUAL score and total read depth (score, depth). Three unique call loci from one of the FreeBayes, GATK, or SNVer were filtered in other variant calling tools

Lastly we detected the SNP concordance of two different mapping tools (BWA-mem and Bowtie2) with the same variant calling tool and the same data preprocess. The different mapping tools impacted the variant calls with only around 50% concordance (**Supplementary Fig.** **S2**).

### Discordance among different variant calling tools

The discordance calls of the seven variant calling tools were first explored by those calls that were missed in one variant calling tool but appeared in all other six tools. As shown in Table 2, VarScan and VarDict missed the most SNP calls, followed by Platypus and SNVer. The FreeBayes, GATK and Samtools/mpileup tools had the lowest number of missed calls in all different mapping tools and differentially preprocessed reads. Interestingly, Samtools/mpileup had fewer missed calls in the BWA-mem alignment than in Bowtie2, which was opposite to FreeBayes and GATK that had fewer missed calls in Bowtie2 than in BWA-mem. We examined the number of reads that supported the variant calls and the quality at those missed call sites. Some missed SNP calls by GATK and Samtools/mpileup were found to not really be missed but due to a stringent filtering instead. For example, the locus 397,560,208 on chromosome 7D (chr7D397560208, Table 2) with QUAL of 29 and total read depth of 4 was filtered out (TR = 2). Similarly, the locus chr2A36897168 was filtered out by Samtools/mpileup. However, one of the five variant calling tools (FreeBayes, Platypus, Snver, VarDict, or VarScan) missed some SNP calls at loci where other tools identified high QUAL and TRs. All these data suggest that GATK and Samtools/mpileup missed fewer true SNPs than other variant calling tools.

We also inspected those calls that appeared in only one variant calling tool out of the seven tools. This examination reflects the sensitivity of each tool, although high sensitivity could mean a higher false positive rate. FreeBayes, VarScan, and VarDict were most sensitive with many unique calls (Table 2). GATK, Platypus, and Snver had the lowest sensitivity with fewest unique calls. Samtools/mpileup was moderately sensitive. The total variant-containing reads (or TRs) and the variant call QUAL were defined based on different variant calling tools’ algorithms, which use different filtering criteria. For example, at site chr1A23072417, FreeBayes identified all 4 aligned reads as TR with a total QUAL score of 111, while GATK and Samtools/mpileup only defined 2 TRs. The four other variant calling tools called nothing at this site. At site chr1A286123173, Samtools/mpileup called four TRs and six other tools called nothing at this site.

### Performance comparison

**Fig. 5:**
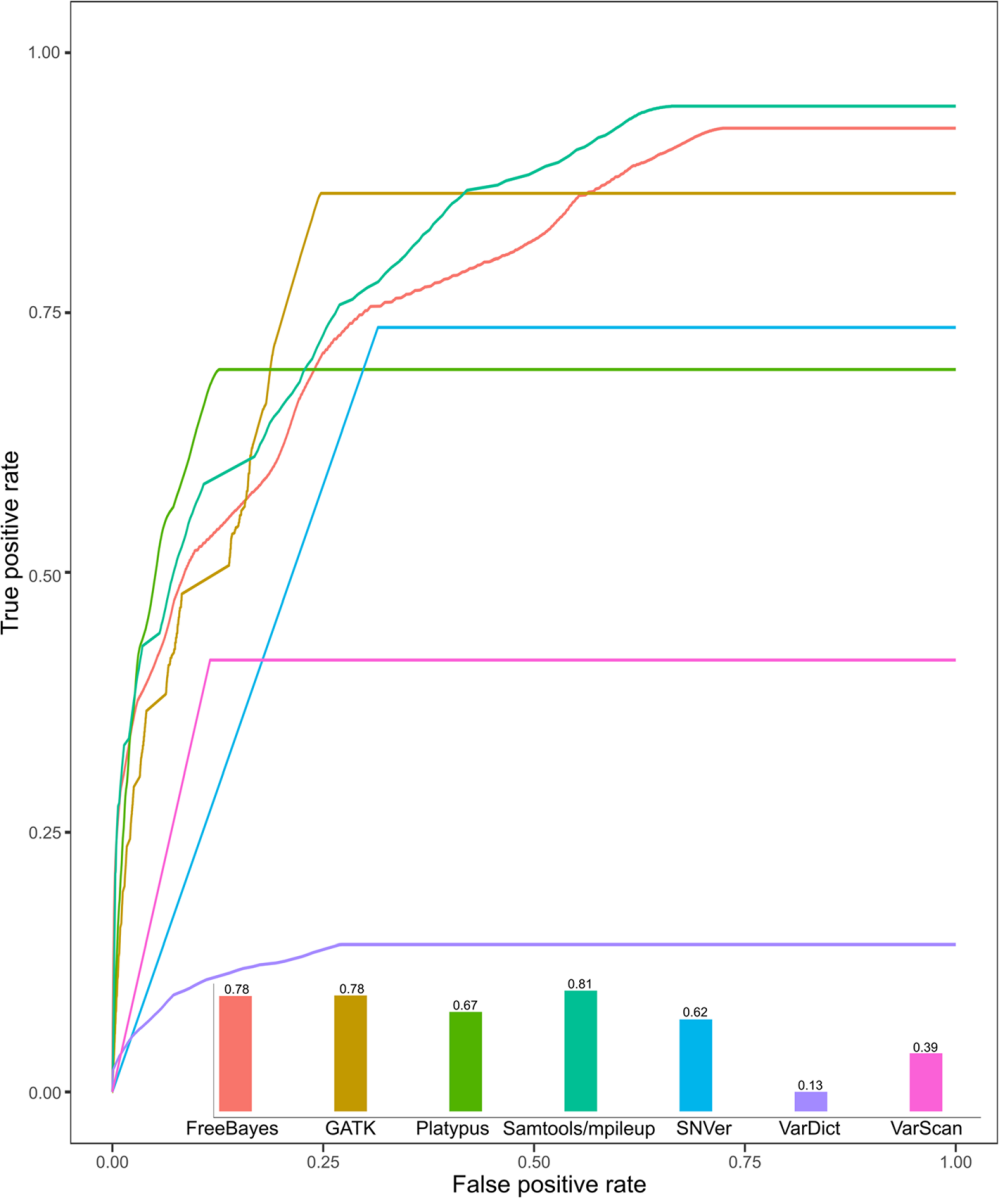
Performance comparison of seven variant calling tools. The performance of a variant calling tool was examined by ROC using the same **mapping tool** (BWA-mem) and the raw read data

We defined a list of “true” SNP calls that were supported by at least 13 out of the 42 experiments with different mapping tools, preprocesses and variant calling tools. The cut-off of 13 experiments was determined by three categories of variant calling tools (Table 1) because each tool detected six datasets and 13 experiments is the worst case for having three tools (6 + 6 + 1 = 13). A total of 505,286 SNPs were used to generate receiver operating characteristic (ROC) curves. The Area Under Curve (AUC) was employed for performance comparison of variant calling tools (**Supplementary Fig.** **S3**). Platypus caught around 60% of true positives if the false positive was controlled under 5%. GATK caught around 90% of true positives under 25% of false positive. However Samtools/mpileup had the best overall AUC (0.81) when using raw reads with the BWA-mem mapping tool, followed by GATK (0.781) and FreeBayes (0.777). The lowest was VarDict (0.13) followed by VarScan (0.39) (Fig. 5**)**.

The performance of different mapping tools in variant detection was examined by using Samtools/mpileup and GATK tools. BWA-mem mapping tool was found to outperform Bowtie2 using either Samtools/mpileup or GATK (**Supplementary Fig.** **S4****)**. The preprocessed data by removing duplicate reads did not improve SNP calling while quality trimming had almost the same AUC performance as the non-preprocessed raw read data (**Supplementary Fig.** **S5****)**.

### Exploring the genetic population diversity in 114 genotypes

**Fig. 6:**
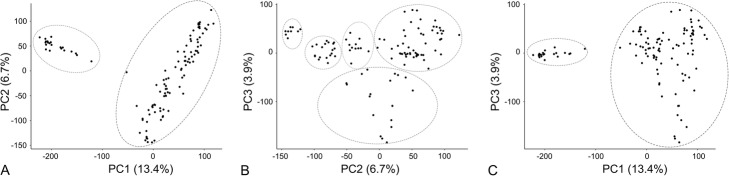
Inspection of genetic variants in a large wheat data set. Whole exome capture sequences of 114 wheat lines were used for SNP calling by Samtools/mpileup. A total of 1,524,455 filtered SNPs were applied to principle component analysis (PCA). The top three PCs were used to project the population genetic variation structure

We applied our best data process (raw reads), mapping tool (BWA-mem), and variant calling tool (Samtools/mpileup) to a larger wheat WEC data set. In total 128,850,093 variants including SNPs and INDELs were called from 114 genotypes. After filtering with a criteria of SNPs only, total variant-containing reads of 10 or greater, and missing calls less than 5% in 114 samples, a total of 1,524,455 SNPs were identified. These SNPs were used to assess the population diversity of the 114 wheat genotypes that represent origins across the whole of Canada. The 114 genotypes were separated into two large groups by principal component 1 (PC1) and PC2 (Fig. 6a). However, additional subgroups were revealed by PC2 and PC3 (Fig. 6b). The structural variances of the same two groups were caught by PC3 and PC1 (Fig. 6c).

## Discussion

In this study we attempted to select a set of genome wide variant analysis procedures that are suitable for wheat or other polyploid crops. Variant calling involves three basic steps: read data pre-processing, read mapping, and variant calling. Thus, we focused on the two widely employed open-source mapping tools Bowtie2 and BWA-mem on three preprocessed read sets, since both the genome and the read data may affect the short sequence reads mapping [13]. Based on read mapping results, we evaluated seven different variant calling tools. As such, we performed variant calling comparisons of 42 combinations at three different levels, i.e., three data preprocesses, two mapping tools, and seven variant calling tools.

Previous studies have comprehensively compared many mapping tools [13–19]. Instead, we only focused on the two most popular mapping tools, BWA-mem and Bowtie2, but expanded our knowledge of the impact of data preprocessing on read mapping, and subsequent variant calling. With the default option settings, BWA-mem mapped more reads than Bowtie2, despite a few multiple mapping sites. BWA-mem also had more accurate mapping rates based on properly paired-end reads mapped and had more mapped reads pass the quality threshold than Bowtie2, indicating BWA-mem is more accurate than Bowtie2 in mapping polyploid wheat genome sequence reads. Though either variant calling tool can be applied in crop genome alignment, our results suggested that BWA-mem was a more suitable mapping tool than Bowtie2 for polyploid wheat genome re-sequence data. In our study, we could not see any benefit to the extra computing efforts in preprocessing the sequence data. The trimming by base quality did not improve the mapping performance of both BWA-mem and Bowtie2. Similar results were previously reported that the adaptor removal did not improve the read mapping [38]. The mapping process itself acts as a quality control in which only good reads can be properly mapped on a reference. The soft clipping process in BWA-mem mapping will remove the unmapped end sequence bases including adaptor or uncertainly called bases, like a quality trimming.

The low concordances of variant calls among different variant calling tools were believed to be attributed by the different intrinsic algorithms of these variant calling tools, data platforms, variant filtering methods, as well as the number of variant calling tools compared in experiments [35]. The call QUAL or read depth alone are inappropriate for SNP filtering. Our filter was based on the total number of reads containing this variant (TR) and their average call QUAL, i.e., we kept a SNP site call that is highly supported by at least three TRs [39]. We could use this same filtering for all seven different variant calling tools. With this filtering we saw a low concordance among the seven variant calling tools though our filtering criteria may look less rigorous. But more stringent filtering could lead to even less overlap. Therefore this low concordance may be attributed to a large difference among these variant calling tools. Comparison of only two or three variant calling tools resulted in more overlaps. It is worth noting that in these tool comparisons, a common practice of using the default settings/parameters was applied to reduce the complexity. For a particular algorithm, changing the parameters, such as twisting the hyper-parameters, usually resulted in different outcomes and eventually better results. However, the intrinsic algorithm appropriate to the genome complexity impacts the performance and accuracy the most.

At a particular genomic locus where the same sequence reads were aligned, it is interesting to explore why some variant calling tools called a SNP while others did not. One reason is that a variant calling tool’s algorithm could not catch this site or this site was filtered out by an inappropriate filtering setting. VarScan and VarDict tools manifested large discordance calls compared to the other five variant calling tools. Our concordance results showed much lower concordance rates than previous reports with ~ 92% concordance observed among the variant calls by three variant calling tools (GATK ∩ Samtools/mpileup ∩ FreeBayes) [21] and ~ 57% [23] and ~ 70% [35] of concordance levels among variant-calling pipelines. These studies used the gold standard sets that were generated from the same one human individual. Our results implied that the variant calling tools should be selected based on the genome complexity. The site-based allele test method, VarDict, had the best performance in targeted human gene variant calling [40]. The haplotype-based Bayesian model method, FreeBayes, may be too sensitive for the hexaploid wheat genome as it detected the highest number of calls (BWA-mem alignment of raw reads), which contained 472,999 SNPs that were not supported by any of other six variant calling tools. Our data indicated that the site-alignment based genotype likelihood method, Samtools/mpileup, is moderate in both specificity and sensitivity, which balanced sensitivity with avoiding being excessively conservative when calling variant bases in the hexaploid wheat genome. Interestingly, in a comparison of four variant calling tools (GATK, Samtools/mpileup, FreeBayes, and Ion Proton Variant Caller TVC), a pipeline with BWA-mem and Samtools/mpileup was also recommended for SNP calling for human WEC sequencing data [21]. The Samtools/mpileup and GATK showed a comparable performance in plant variant calling [22].

For an accuracy comparison of different variant calling tools, the real or “true” positive call data is required to estimate the TPR and FPR of different calling tools. Although a gold standard of variant datasets has been developed for evaluating human variant calling tools [34], there is no gold standard of datasets available for crop variant calling tool evaluation. In an attempt to benchmark variant identification tools for plant diversity discovery [22], the main effort has been put on mapping tools. However, only two variant calling tools, GATK and Samtools/mpileup, were evaluated by the total SNP calls from tomato data sets instead of by TPR because the true positives were unknown, though simulation data has been used to conduct the Precision Recall analysis in this study [22]. We defined a “true” positive SNP list as those calls that were supported by at least 13 out of 42 experiments of different preprocessed data, mapping tools, and variant calling tools (supported by at least three variant calling tools). These “true” positive list allowed us to measure the performance of variant calling tools using an area under the ROC curve. Samtools/mpileup showed the best overall performance followed by GATK. The VarScan and VarDict do not appear to work well for polyploid crop genomes. Consistent with the concordance data, the ROC results also supported the Samtools/mpileup as a suitable variant calling tool for hexaploid wheat.

When we applied the procedure of mapping with BWA-mem and variant calling with Samtools/mpileup to the data of the 114 lines, we found 1,524,455 SNPs within the exome of allohexaploid wheat that could be used for diversity analysis. Two main diversity groups and several subgroups were revealed by the top three PCs from principal component analysis which explained 13.4%, 6,7, and 3.8% of variance of the population, with respect to PC1, PC2, and PC3, indicative of effectiveness for a large SNP data set. For example, in a human study, the first two components represented only 0.3693 and 0.117% of the variation yet revealed clear population structure in a large dataset with over 107,000 SNPs for over 6000 people [41]. In contrast, a PCA graph could capture a large percentage of the total variation, even 50% or more, but that would not guarantee that it will show evident structure in the data [42]. Our SNP discovery in this population data collection would help in investigating trait-associated variants by high functional impacts.

A limitation of the current study is our focus on SNPs and not evaluating these tools for INDEL detection. The decision to consider only SNPs was based on the following considerations: 1) Due to their high frequency and binary variation patterns, SNPs are most interesting as generic markers in various biological studies. However, INDELs are not; 2) INDELs are the hardest to detect among variants, for example, because of the different lengths of INDELs. A variant calling tool may have a different strength for SNP and INDEL detection. In order to make a clean analysis and lead to a clear conclusion, we decided not to include INDEL data in the current study.

## Conclusion

In summary, for the complex wheat genome our recommendation is to use a BWA-mem and Samtools/mpileup pipeline for SNP calling. This would be a good starting point for other polyploid crop species. There is no need to preprocess the raw read data before mapping onto a reference genome. A recommended SNP filtering is at least 3 reads containing the variant with average QUAL of at least 5. This filtering can be more stringent depending on the needs of the specific study. Our study will provide a practical and comprehensive guide to more accurate and consistent variant identification, ultimately leading to crop genome variant information for breeding, diversity study, and germplasm genotyping.

## Methods

### Sequence data and experiment design

As an initial methodology study, the unpublished wheat allohexaploid WEC sequence data generated by the Canadian Triticum Applied Genomics (CTAG2) was used. Briefly, 114 diverse hexaploid wheat lines representing the genetic diversity of a large wheat population in Canada were collected. Genomic DNA was extracted from leaf tissue for each accession using the Agencourt DNAdvance Genomic DNA Isolation Kit (Beckman Coulter) and subjected to sequence capture using the NimbleGen SeqCap EZ wheat whole-genome assay to generate a sequence capture library [3]. The sequence capture library was sequenced on an Illumina HiSeq2000 instrument to generate about 30 million reads per accession. Three data sets (raw read fastq file, preprocessed with quality trimming, and duplicate removal) generated from one sample were used for variant calling tool evaluation before a whole population diversity was investigated. The experiment design and workflow were depicted in Fig. 1.

### Sequence data preprocess

Besides the raw read fastq files, two preprocessed fastq files were produced from the same sample. The software Prinseq-lite v0.20.4 [43] with options -trim_qual_right 10 and -trim_qual_left 10 to generate trim fastq file. These options trimmed off the bases from both ends of reads that meet the first base call quality less than 10. Prinseq-lite with an additional option derep 1 created rep1 fastq in which the duplicates, and end bases with poor quality were removed.

### Sequence mapping

The sequence reads were mapped to the wheat reference genome (IWGSC RefSeq v1.0). Two mapping tools BWA-mem (v0.7.17) [25] and Bowtie2 (v2.3.4.3) [26] were run with their default parameters (Table 1). BWA-mem mapper had default settings of penalty 4 for a mismatch, gap open penalties 6 and 6 for deletions and insertions. BWA-mem conducted for 5′- and 3′-end clipping both with penalty of 5. BWA-mem reported multiple mapped reads in bam files. Bowtie2 had a maximum penalty 6 for a mismatch, and gap open and extend penalties of 5 and 3, respectively. Bowtie2 only reported the best mapping quality (MQ) read for multiple alignments. For mapping statistics such as the total mapped, unmapped, and paired mapped reads, and further analysis of the alignment files, Samtools v1.9 [44] was employed.

### Variant calling tools, parameters and filtering

Seven variant calling tools, their description and parameters are summarized in **Table** 1 . All vcf files of the seven tools were filtered by QUAL/TR > 5 and TR > 2 or the calls with TR > 40.

### Concordance of the seven variant calling tools

For concordance analysis, variants identified with different data preprocessing and mapping tool were merged into one VCF file for each variant calling tool. The merged VCF was filtered against low confident variants with the same filtering criteria as described above. Venn diagrams and percentage of overlap were generated to examine the concordance among the seven variant calling tools under different mapping tools and data preprocesses.

### ROC curves

The vcflib-1.0.1 package [45] was used to calculate the true positive rate (TPR) and false positive rate (FPR) of each variant calling tool and generate ROC curves. The input true positives were the SNPs that were supported by at least three variant calling tools or 13 out of the 42 calling experiments. The cut-off of three variant calling tools was based on the three classes of algorithms (Table 1): haplotype-based (FreeBayes, GATK, Platypus), site align-based likelihoods (Samtools/mpileup, SNVer), and site-based allele frequency (VarScan, VarDict). Thirteen experiments represented the worst case for having three tools since each variant calling tool detected 6 datasets. The ROC curves were created by the vcfroc program in the same vcflib package. Vcfroc conducted TPR and FPR calculations as follows:

TPR = TP/(TP + FN), where TP is the true positive number, FN the false negative number.

FPR = FP/(FP + TN), where FP is the false positives, TN the true negatives.

### Principle component analysis (PCA)

The vcf generated from 114 wheat lines by the BWA-mem mapping tool and Samtools/mpileup variant calling tool was filtered with a criteria of SNPs only, total variant-containing reads (TR) of10 or greater, and missing calls less than 5% in 114 samples. The filtered vcf file generated from variant calling of 114 wheat lines was entered into TASSEL v5 package [46] for PCA analysis. The genotype calls were transformed and eigenvalue of each variable was calculated by variance correlation and transformation. The top three components’ eigenvalues were exported for PCA plot using R script.

## Supplementary information

**Additional file: Supplementary material file: detailed information. Supplementary Fig. S1.** Venn diagrams for SNP calls on different preprocessed read sets. **Supplementary Fig. S2.** Venn diagram for SNP calls on differently mapped reads. **Supplementary Fig. S3.** Receiver operating characteristic curve (ROC) comparison of variant calling tools. **Supplementary Fig. S4.** Performance comparison of read mapping tools in terms of variant calling. **Supplementary Fig. S5.** Performance comparison of data preprocesses in terms of variant calling. **Supplementary Table S1.** Statistics of short read mapping by different mapping tools. **Supplementary Table S2.** Numbers of SNP calls of seven variant calling tools with default and post-filtering criteria. **Supplementary Data 1.** Data_1_FreeBayes.6.f.vcf.gz. Six VCF files generated by FreeBayes calling tool from alignment data of three sets (raw, trim, and rep1) and two mapping tools (BWA-mem and Bowtie2). **Supplementary Data 2.** Data_2_GATK.6.f.vcf.gz. Six VCF files generated by GATK calling tool from alignment data of three sets (raw, trim, and rep1) and two mapping tools (BWA-mem and Bowtie2). **Supplementary Data 3.** Data_3_Platypus.6.f.vcf.gz. Six VCF files generated by Platypus calling tool from alignment data of three sets (raw, trim, and rep1) and two mapping tools (BWA-mem and Bowtie2). **Supplementary Data 4.** Data_4_Samtools.6.f.vcf.gz. Six VCF files generated by Samtools/mpileup calling tool from alignment data of three sets (raw, trim, and rep1) and two mapping tools (BWA-mem and Bowtie2). **Supplementary Data 5.** Data_5_SNVer.6.f.vcf.gz. Six VCF files generated by SNVer calling tool from alignment data of three sets (raw, trim, and rep1) and two mapping tools (BWA-mem and Bowtie2). The VCF file has been filtered by criteria described in text. **Supplementary Data 6.** Data_6_VarDict.6.f.vcf.gz. Six VCF files generated by VarDict calling tool from alignment data of three sets (raw, trim, and rep1) and two mapping tools (BWA-mem and Bowtie2). **Supplementary Data 7.** Data_7_VarScan.6.f.vcf.gz. Six VCF files generated by VarScan calling tool from alignment data of three sets (raw, trim, and rep1) and two mapping tools (BWA-mem and Bowtie2).

## Data Availability

The data (VCFs) generated during this study and supporting the conclusions are included in this published article and its supplementary information files. The large datasets tested only during the current study but generated by a previous project are available from the authors on reasonable request.

## Abbreviations

WGS: Whole genome sequencing
WEC: Whole exome capture sequencing
GBS: Genotyping-by-sequencing
ROC: Receiver operating characteristic curve
AUC: Area under the curve
MQ: Mapping quality score
SNP: Single nucleotide polymorphism
GATK: Genome analysis tool kit
PCA: Principal component analysis
GIAB: Genome in a bottle consortium
NGS: Next generation sequencing
QUAL: Variant call quality score
TPR: True positive rate
FPR: FALSE positive rate
TR: Total number of reads containing this variant
CTAG2: Canadian triticum applied genomics
