# Supplementary material for: Evaluation of variant calling tools for large plant genome re-sequencing
Source: BMC Bioinformatics. 2020 Aug 17;21:360. doi: 10.1186/s12859-020-03704-1 (PMC7430858; doi:10.1186/s12859-020-03704-1)
Supplement: Supplementary file 1 — Additional file: Supplementary material file: detailed information. Supplementary Fig. S1. Venn diagrams for SNP calls on different preprocessed read sets. Supplementary Fig. S2. Venn diagram for SNP calls on differently mapped reads. Supplementary Fig. S3. Receiver operating characteristic curve (ROC) comparison of variant calling tools. Supplementary Fig. S4. Performance comparison of read mapping tools in terms of variant calling. Supplementary Fig. S5. Performance comparison of data preprocesses in terms of variant calling. Supplementary Table S1. Statistics of short read mapping by different mapping tools. Supplementary Table S2. Numbers of SNP calls of seven variant calling tools with default and post-filtering criteria. Supplementary Data 1. Data_1_FreeBayes.6.f.vcf.gz. Six VCF files generated by FreeBayes calling tool from alignment data of three sets (raw, trim, and rep1) and two mapping tools (BWA-mem and Bowtie2). Supplementary Data 2. Data_2_GATK.6.f.vcf.gz. Six VCF files generated by GATK calling tool from alignment data of three sets (raw, trim, and rep1) and two mapping tools (BWA-mem and Bowtie2). Supplementary Data 3. Data_3_Platypus.6.f.vcf.gz. Six VCF files generated by Platypus calling tool from alignment data of three sets (raw, trim, and rep1) and two mapping tools (BWA-mem and Bowtie2). Supplementary Data 4. Data_4_Samtools.6.f.vcf.gz. Six VCF files generated by Samtools/mpileup calling tool from alignment data of three sets (raw, trim, and rep1) and two mapping tools (BWA-mem and Bowtie2). Supplementary Data 5. Data_5_SNVer.6.f.vcf.gz. Six VCF files generated by SNVer calling tool from alignment data of three sets (raw, trim, and rep1) and two mapping tools (BWA-mem and Bowtie2). The VCF file has been filtered by criteria described in text. Supplementary Data 6. Data_6_VarDict.6.f.vcf.gz. Six VCF files generated by VarDict calling tool from alignment data of three sets (raw, trim, and rep1) and two mapping tools (BWA-mem and Bowtie2). Supplem [file 12859_2020_3704_MOESM1_ESM.pdf]

Evaluations of variant calling tools for large plant genome re-sequencing

Zhen Yao[1], Frank M. You[2], Amidou N'Diaye[3]. Ron E. Knox[4], Curt McCartney[1], Colin W. Hiebert[1], Curtis Pozniak[3], Wayne Xu[1]*

Morden Research and Development Centre, Agriculture and Agri-Food Canada, 101 Route 100, Morden, Manitoba R6M 1Y5, Canada,

[2]Ottawa Research and Development Centre, Agriculture and Agri-Food Canada, 960 Carling Avenue, Ottawa, Ontario K1A 0C6, Canada

[3]Department of Plant sciences, University of Saskatchewan, Saskatoon, SK S7N 5A8, Canada,

[4]Swift Current Research and Development Centre, Agriculture and Agri-Food Canada, Box 1030 Swift Current, Saskatchewan S9H 3X2, Canada

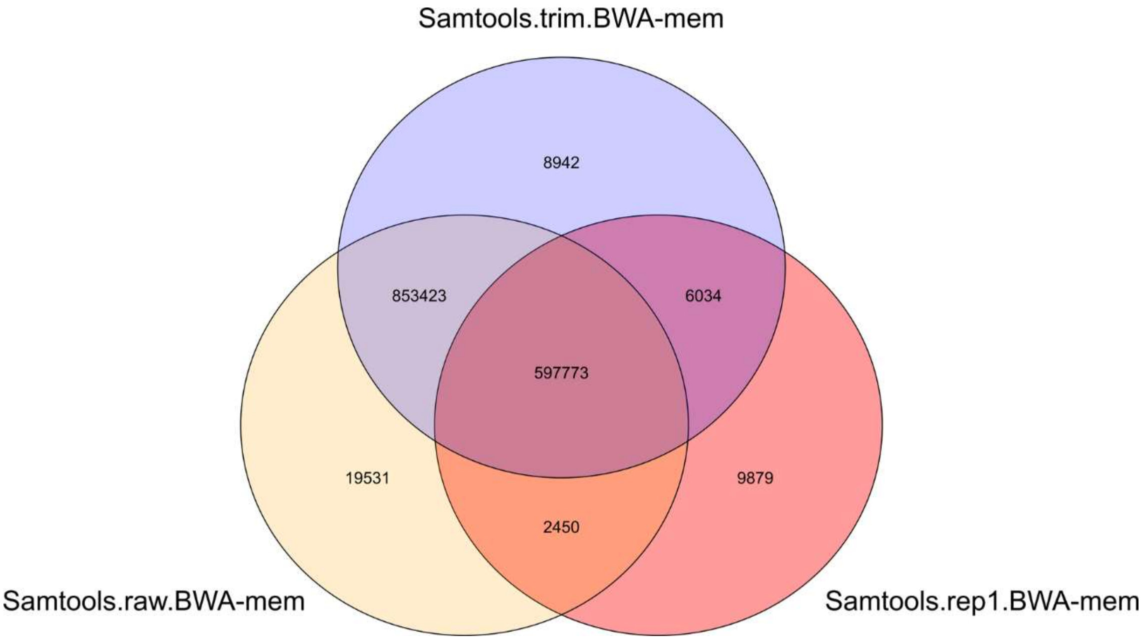

Supplemental Fig S1. Venn diagram for SNP calls on different preprocessed read sets. The raw sequence reads, quality trimmed reads, or the duplicate removed reads were mapped on reference genome by BWA-mem mapping tool. SNP variants were called by samtool and passed through the same stringent filtering.

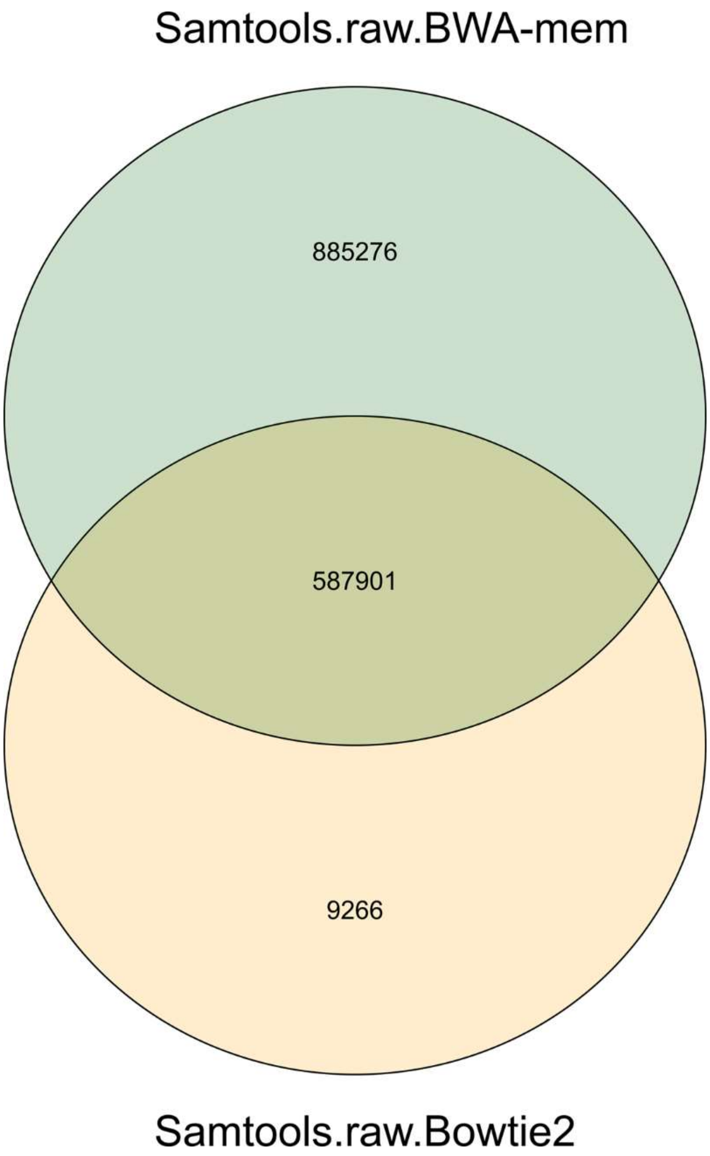

Supplemental Fig S2. Venn diagram for SNP calls on differently mapped reads. The raw reads were mapped on reference genome by either BWA-mem or Bowtie2. SNP variants were called by Samtools/mpileup and passed through the same stringent filtering.

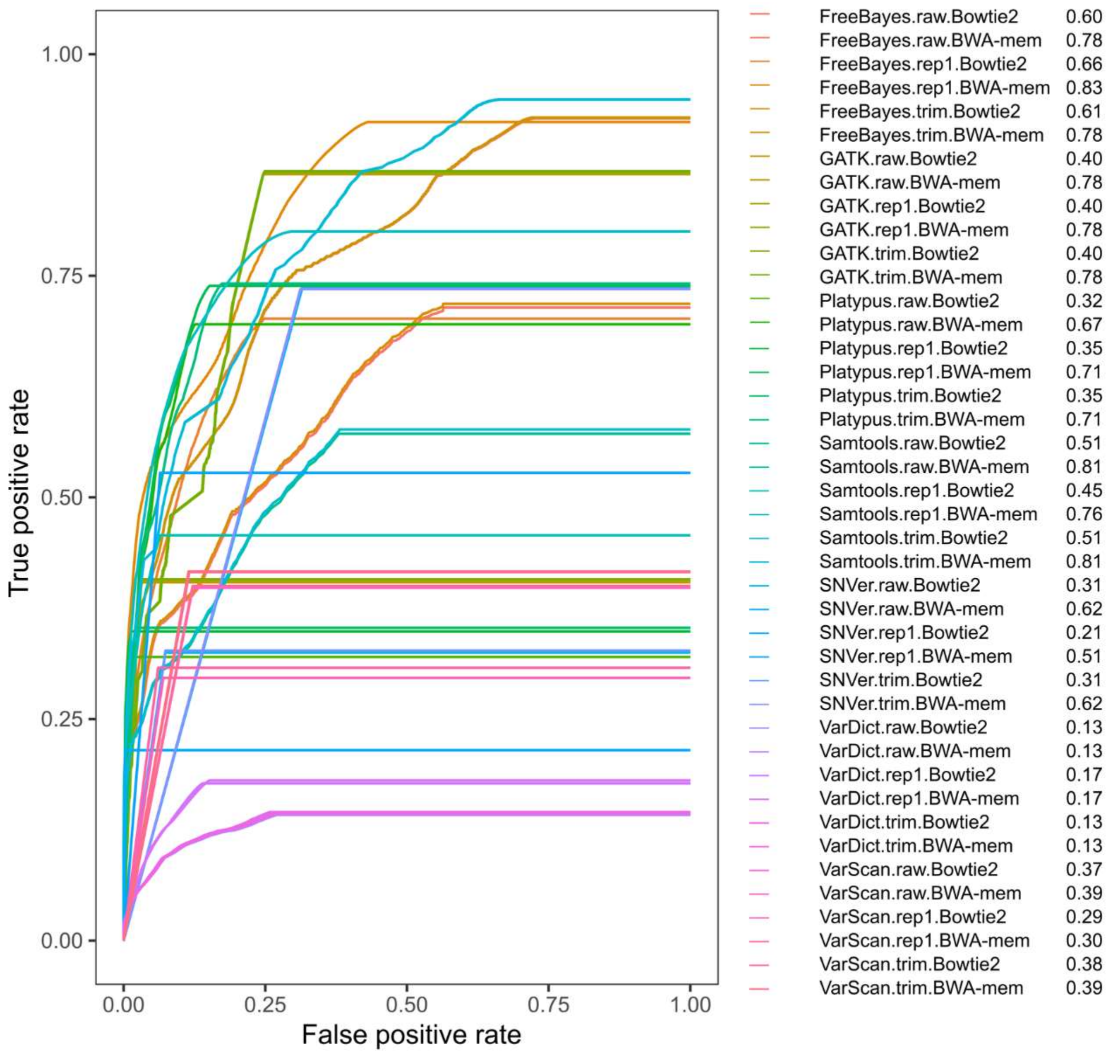

Supplemental Fig S3. Receiver operating characteristic curve (ROC) comparison of variant calling tools in 42 combinations of read preprocessing methods, mapping tools and variant calling tools. The real positive SNP calls were defined by those SNPs that were supported by at least three variant calling tools or by 13 out of 42 tests. The True Positive Rate (TPR) and False Positive Rate (FTR) of each of the 42 tests were calculated and the ROCs were plotted by vcflib package. The Area Under Curve (AUC) is labeled on the right panel. The higher the AUC the better the performance.

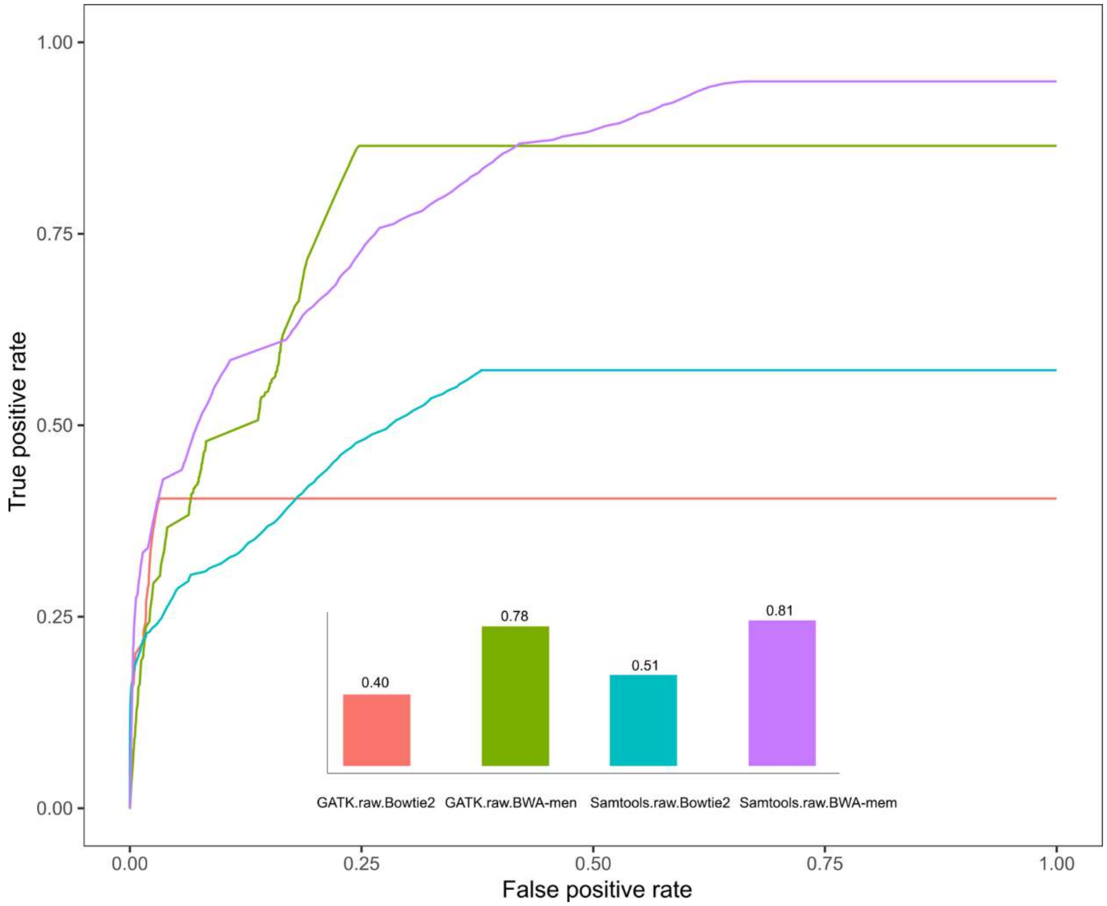

Supplemental Fig S4. Performance comparison of read mapping tools in terms of variant calling. The performances of BWA-mem and Boutie2 were compared by ROC either using Samtools/mpileup or GATK variant calling tool. Raw read sets were used in these comparisons.

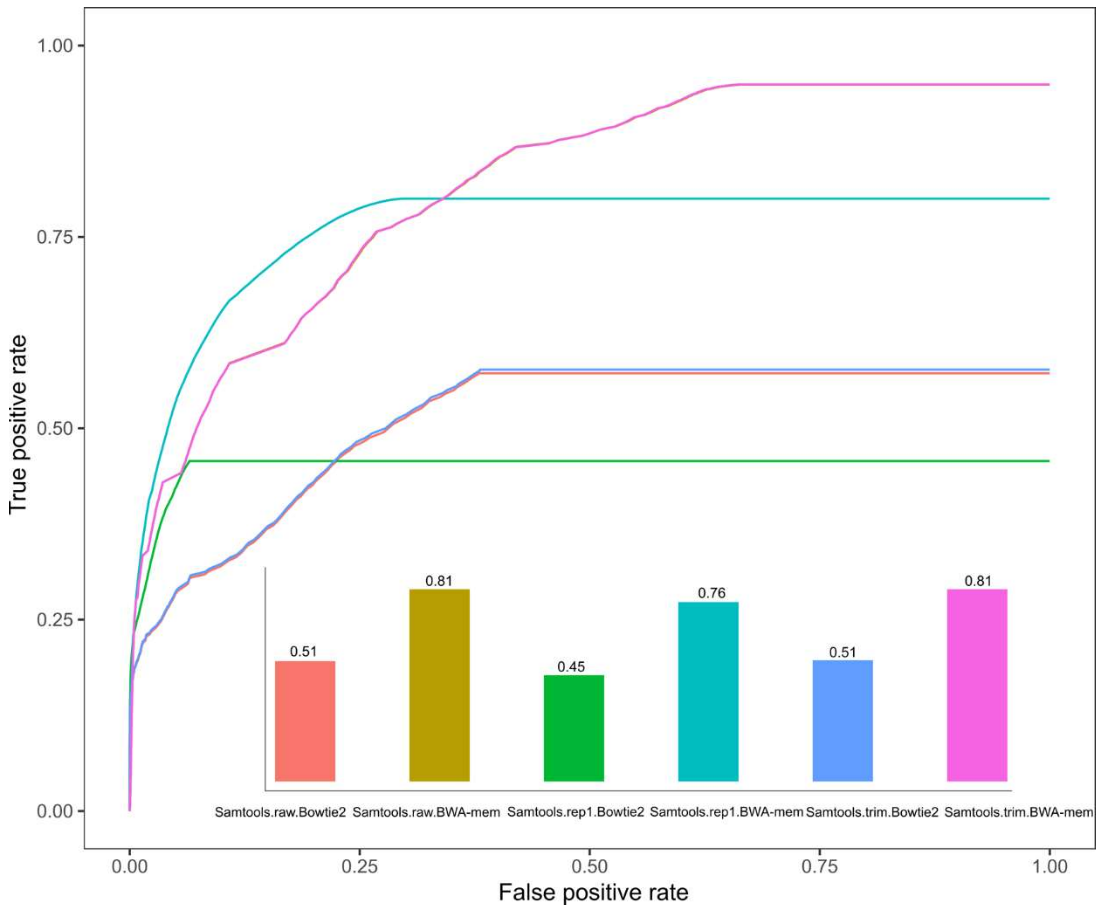

Supplemental Fig S5. Performance comparison of data preprocesses in terms of variant calling. The performance of raw reads, quality trimming, or duplicate removal was compared by ROC in variant calling. Variant calling tool Samtools/mpileup and read mapping tool BWA-mem were used in these comparisons.

Supplemental Table S1. Statistics of short read mapping by different mapping tools.

		Bowtie2			BWA-mem		
		raw	trim	rep1	raw	trim	rep1
fastq read#		97280936	96718760	70099964	97280936	96718760	70099964
F4		95230913	94966314	68755954	97378651	96770306	70094900
	%	97.9	98.2	98.1	100.1	100.1	100.0
f4		2050023	1752446	1344010	236364	264001	243635
	%	2.1	1.8	1.9	0.2	0.3	0.3
f2		91961762	91927288	66566384	95783813	95244789	68852224
	%	94.5	95.0	95.0	98.5	98.5	98.2
MQ10		62305341	62310926	45247503	78481028	77949625	56526810
	%	64.0	64.4	64.5	80.7	80.6	80.6
mm10+		62229033	62283313	45226944	47531812	47206286	34437070
	%	64.0	64.4	64.5	48.9	48.8	49.1

*F4, f4, and f2 are Samtools flags for extraction of mapped, unmapped, and properly paired mapped reads, respectively. MQ10 represents the number of reads with mapping quality of 10 and above. Mm10+ means a filter of MQ10 and mismatched bases per sample less than 10.

Supplemental Table S2. Numbers of SNP calls of seven variant calling tools with default and post-filtering criteria.

		BWA-mem			Bowtie2		
		raw	trim	rep1	raw	trim	rep1
FreeBayes	calls	5112707	4971478	3573595	5968773	5734169	4062404
	filtered	1792861	1774696	850475	1016524	1017579	521039
GATK	calls	2450932	2455787	2460139	901056	905099	910982
	filtered	603427	606317	605966	221109	223086	222539
Platypus	calls	608553	748603	735801	235403	298241	292451
	filtered	424859	481143	463743	166063	190355	183465
SNVer	calls	604292	600981	301474	204407	205720	110140
	filtered	604293	600982	301475	204408	205721	110141
Samtools	calls	6787638	6760894	6359663	2175286	2188534	1580346
	filtered	1473177	1466172	616136	597167	602283	266123
VarScan	calls	276452	275904	188340	271495	272234	187955
	filtered	276453	275905	188341	271496	272235	187956
VarDict	calls	737886	738676	592434	715421	717204	572073
	filtered	261178	261818	182039	247735	249078	172364

*Three levels of factors were examined in variant calling, preprocessed read sets (raw, trim, and rep1), mapping tools (BWA-mem and Bowtie2), and variant calling tools (FreeBayes, GATK, Samtools/mpileup, Platypus, SNVer, VarDict, and VarScan).

Supplemental Data 1. Data_1_FreeBayes.6.f.vcf.gz.

Six VCF files were generated by FreeBayes calling tool from alignment data of three sets (raw, trim, and rep1) and two mapping tools (BWA-mem and Bowtie2). The VCF file has been filtered by criteria described in text.

Supplemental Data 2. Data_2_GATK.6.f.vcf.gz.

Six VCF files were generated by GATK calling tool from alignment data of three sets (raw, trim, and rep1) and two mapping tools (BWA-mem and Bowtie2). The VCF file has been filtered by criteria described in text.

Supplemental Data 3. Data_3_ Platypus.6.f.vcf.gz.

Six VCF files were generated by Platypus calling tool from alignment data of three sets (raw, trim, and rep1) and two mapping tools (BWA-mem and Bowtie2). The VCF file has been filtered by criteria described in text.

Supplemental Data 4. Data_4_ Samtools.6.f.vcf.gz.

Six VCF files were generated by Samtools/mpileup calling tool from alignment data of three sets (raw, trim, and rep1) and two mapping tools (BWA-mem and Bowtie2). The VCF file has been filtered by criteria described in text.

Supplemental Data 5. Data_5_SNVer.6.f.vcf.gz.

Six VCF files were generated by SNVer calling tool from alignment data of three sets (raw, trim, and rep1) and two mapping tools (BWA-mem and Bowtie2). The VCF file has been filtered by criteria described in text.

Supplemental Data 6. Data_6_ VarDict.6.f.vcf.gz.

Six VCF files were generated by VarDict calling tool from alignment data of three sets (raw, trim, and rep1) and two mapping tools (BWA-mem and Bowtie2). The VCF file has been filtered by criteria described in text.

Supplemental Data 7. Data_7_ VarScan.6.f.vcf.gz.

Six VCF files were generated by VarScan calling tool from alignment data of three sets (raw, trim, and rep1) and two mapping tools (BWA-mem and Bowtie2). The VCF file has been filtered by criteria described in text.
